# Admission factors associated with hospital mortality in patients with haematological malignancy admitted to UK adult, general critical care units: a secondary analysis of the ICNARC Case Mix Programme Database

**DOI:** 10.1186/cc8016

**Published:** 2009-08-25

**Authors:** Peter A Hampshire, Catherine A Welch, Lawrence A McCrossan, Katharine Francis, David A Harrison

**Affiliations:** 1 Royal Liverpool University Hospital, Prescot Street, Liverpool, L7 8XP, UK; 2 Intensive Care National Audit and Research Centre, Tavistock House, Tavistock Square, London, WC1H 9HR, UK; 3 Milton Keynes Hospital NHS Foundation Trust, Standing Way, Eaglestone, MK6 5LD, UK

## Abstract

**Introduction:**

Patients with haematological malignancy admitted to intensive care have a high mortality. Adverse prognostic factors include the number of organ failures, invasive mechanical ventilation and previous bone marrow transplantation. Severity-of-illness scores may underestimate the mortality of critically ill patients with haematological malignancy. This study investigates the relationship between admission characteristics and outcome in patients with haematological malignancies admitted to intensive care units (ICUs) in England, Wales and Northern Ireland, and assesses the performance of three severity-of-illness scores in this population.

**Methods:**

A secondary analysis of the Intensive Care National Audit and Research Centre (ICNARC) Case Mix Programme Database was conducted on admissions to 178 adult, general ICUs in England, Wales and Northern Ireland between 1995 and 2007. Multivariate logistic regression analysis was used to identify factors associated with hospital mortality. The Acute Physiology and Chronic Health Evaluation (APACHE) II score, Simplified Acute Physiology Score (SAPS) II and ICNARC score were evaluated for discrimination (the ability to distinguish survivors from nonsurvivors); and the APACHE II, SAPS II and ICNARC mortality probabilities were evaluated for calibration (the accuracy of the estimated probability of survival).

**Results:**

There were 7,689 eligible admissions. ICU mortality was 43.1% (3,312 deaths) and acute hospital mortality was 59.2% (4,239 deaths). ICU and hospital mortality increased with the number of organ failures on admission. Admission factors associated with an increased risk of death were bone marrow transplant, Hodgkin's lymphoma, severe sepsis, age, length of hospital stay prior to intensive care admission, tachycardia, low systolic blood pressure, tachypnoea, low Glasgow Coma Score, sedation, PaO_2_:FiO_2_, acidaemia, alkalaemia, oliguria, hyponatraemia, hypernatraemia, low haematocrit, and uraemia. The ICNARC model had the best discrimination of the three scores analysed, as assessed by the area under the receiver operating characteristic curve of 0.78, but all scores were poorly calibrated. APACHE II had the highest accuracy at predicting hospital mortality, with a standardised mortality ratio of 1.01. SAPS II and the ICNARC score both underestimated hospital mortality.

**Conclusions:**

Increased hospital mortality is associated with the length of hospital stay prior to ICU admission and with severe sepsis, suggesting that, if appropriate, such patients should be treated aggressively with early ICU admission. A low haematocrit was associated with higher mortality and this relationship requires further investigation. The severity-of-illness scores assessed in this study had reasonable discriminative power, but none showed good calibration.

## Introduction

Patients with haematological malignancies can now expect a greater chance of curative treatment and longer survival times than ever before due to bone marrow (haemopoeitic stem cell) transplantation and chemotherapy. Yet these potentially life-saving treatments may also cause life-threatening complications [1-5]. Seven per cent of patients admitted to hospital with haematological malignancy become critically ill [6], and these patients have a higher mortality than the general intensive care population [7-10].

Factors found to influence survival of patients admitted to the intensive care unit (ICU) with a haematological malignancy include the severity of the acute illness [11-13], invasive mechanical ventilation (IMV) [5,14,15], and previous haemopoeitic stem cell transplant (HSCT) [11,12]. Neutropaenia [12,16] and the nature and progress of the haematological malignancy [9] may also predict a poor outcome. Probably due to the small number of patients included, however, not all of the factors mentioned above were predictive of adverse outcome in subsequent studies.

Models that incorporate the effect of chronic health and specific diagnoses on mortality, such as the Acute Physiology and Chronic Health Evaluation II (APACHE II) score and the Simplified Acute Physiology Score II (SAPS II), are able to discriminate survivors from nonsurvivors [12,16,17]. Despite this ability, severity-of-illness scores significantly underestimate actual mortality in this population of patients [6,8,11]. The Intensive Care National Audit and Research Centre (ICNARC) model was developed in 2007 using data from 216,626 admissions in the ICNARC database [18], and was shown to be superior to existing risk prediction models. The ICNARC model assesses acute physiology in addition to age, source of admission, diagnostic category and cardiopulmonary resuscitation before admission. Unlike the APACHE II and SAPS II models, the ICNARC model does not exclude patients with specific diagnoses, like burns. The model, however, has never been assessed for its accuracy in haematological malignancy patients. The accuracy of a severity-of-illness score can be assessed by the model's discrimination between survivors and nonsurvivors (how well the model predicts the correct outcome) and by assessing calibration (how well the model tracks outcomes across the range of possible scores).

The present study examines the outcomes of haematological malignancy patients admitted to general adult ICUs in England, Wales and Northern Ireland, identified using a high-quality clinical database. We used multivariable logistic regression analysis to identify factors on admission that are associated with acute hospital mortality. We evaluated the discrimination and calibration of the APACHE II, SAPS II and ICNARC models in these patients.

## Materials and methods

### Case Mix Programme Database

The Case Mix Programme is the national comparative audit of adult, general critical care units (ICUs and combined intensive care and high-dependency units) in England, Wales and Northern Ireland, coordinated by the ICNARC. The Case Mix Programme Database (CMPD) contains pooled case mix and outcome data on consecutive admissions to units participating in the Case Mix Programme, which have undergone extensive local and central validation. The data are collected to precise rules and definitions by trained data collectors. Details of the data collection and validation have been reported previously [19]. The CMPD has been independently assessed to be of high quality [20]. Support for the collection and use of patient-identifiable data without consent in the Case Mix Programme has to be obtained under Section 251 of the NHS Act 2006 (approval number PIAG 2–10(f)/2005), and therefore ethical approval was not required for the present study. Data were extracted from the CMPD for 514,918 admissions from 178 ICUs, covering the period December 1995 to March 2007.

### Selection of cases

Admissions in the CMPD with haematological malignancy can be identified from the primary, secondary and ultimate primary reason for admission fields, from either of two other conditions relevant to the admission, and from the past medical history. The reasons for admission and other conditions relevant to the admission are coded using the ICNARC Coding Method [21], a hierarchical method specifically designed for coding reasons for admission to the ICU.

Admissions with any of the following ICNARC Coding Method conditions as the primary, secondary or ultimate primary reason for admission or other conditions relevant to the admission were included in the analysis: bone marrow transplant, graft versus host disease, acute lymphoblastic leukaemia, acute myeloblastic leukaemia, chronic lymphocytic leukaemia, chronic myelogenous leukaemia, Hodgkin's lymphoma, non-Hodgkin's lymphoma or myeloma. All admissions that do not satisfy these criteria but have any of the following conditions in their past medical history were also included in the analysis: acute myelogenous leukaemia or lymphocytic leukaemia or multiple myeloma; chronic myelogenous leukaemia or chronic lymphocytic leukaemia; or lymphoma. The conditions specified above must have been present in the 6 months prior to admission to the unit in order to be included in the CMPD.

An algorithm was derived to divide these admissions into categories based on their reason for admission. This algorithm was required because it is possible for each admission to have more than one condition coded. The following hierarchy of reason for admission was therefore used: acute lymphoblastic leukaemia or acute myeloblastic leukaemia or myeloma; chronic lymphocytic leukaemia or chronic myelogenous leukaemia; Hodgkin's lymphoma or non-Hodgkin's lymphoma; and bone marrow transplant or graft versus host disease.

Each of the reasons for admission or each of the conditions relevant to the admission was searched in turn for the conditions in the order defined above and the admission was allocated to the condition that was identified first. The primary reason for admission was searched first, followed by the secondary reason, the ultimate reason and finally the other conditions relevant to the patient's admission.

It is not possible to identify treatments received by the admitted patient using the ICNARC Coding Method, so admissions with the conditions bone marrow transplant or graft versus host disease were considered to have received HSCT.

### Data

Data were extracted on the case mix, on the outcome and on the activity as defined below.

#### Case mix

Organ system failures were identified from physiological data according to the definitions of Knaus and colleagues [22]. Severity of illness was measured by the APACHE II Acute Physiology Score, the APACHE II score [22], the ICNARC physiology score [18], the SAPS II score [23] and the number of organ system failures. Both the APACHE II Acute Physiology Score and the ICNARC physiology score encompass a weighting for acute physiology (defined by derangement from the normal range for 12 physiological variables in the first 24 hours following admission to the ICU). The APACHE II score and the SAPS II additionally encompass a weighting for age and for a past medical history of specified conditions.

Patients who were ventilated at any time during the first 24 hours in the ICU include both patients who were receiving mechanical ventilation on admission to the ICU and those for which ventilation was initiated at any time during the first 24 hours of their stay.

Patients were defined as having severe sepsis if they met at least three of the four systemic inflammatory response syndrome criteria, if they had evidence of infection, and by the presence of at least one organ dysfunction during the first 24 hours following admission to the ICU. Physiological definitions of the systemic inflammatory response syndrome criteria and organ dysfunctions were matched as closely as possible to those used in the PROWESS trial, as has been reported previously [24].

#### Outcome

Survival data were extracted at discharge from the Case Mix Programme unit and at ultimate discharge from the acute hospital.

#### Readmissions

Readmissions to the unit within the same hospital stay were identified from the postcode, date of birth and sex of the patient, and were confirmed by the participating units.

### Analyses

A statistical analysis plan was agreed *a priori*. The analyses performed were as follows.

#### Descriptive statistics

The case mix, outcome and activity were described for all haematological malignancy admissions.

#### Prognostic modelling in haematological malignancies

The effect of case mix factors on acute hospital mortality was assessed by multivariable logistic regression modelling for the admissions that were identified as having a haematological malignancy. The past medical history as recorded in the CMPD does not distinguish between individual haematological diagnoses, but groups together the following diagnoses: acute myelogenous leukaemia or lymphocytic leukaemia or multiple myeloma; chronic lymphocytic leukaemia or chronic myelogenous leukaemia; and Hodgkin's lymphoma or non-Hodgkin's lymphoma. To assess the effect of specific haematological diagnoses on outcome, therefore, only admissions with a haematological diagnosis as the primary, secondary or ultimate reason for admission were included in the regression analysis of diagnosis on outcome.

For all physiology variables, all measurements were from the first 24 hours following ICU admission. The variables entered into the model, selected *a priori*, were as follows: age; sex; haematological diagnosis on admission (only admissions with a haematological diagnosis as the primary, secondary or ultimate reason for admission were included in this analysis); highest central temperature (or noncentral temperature + 1°C if no central temperature was recorded); lowest systolic blood pressure; highest heart rate; lowest respiratory rate; PaO_2_:FiO_2 _(with additional weightings for patients who were ventilated at any time during the first 24 hours of their admission to the unit); lowest arterial pH; serum sodium (most extreme value from the normal range); serum potassium (most extreme value from the normal range); serum urea (most extreme value from the normal range); serum creatinine (most extreme value from the normal range); urine output in the first 24 hours of admission to the unit (if the length of stay in the unit was less than 24 hours the urine output during their stay is scaled up to give the equivalent urine output in 24 hours); haematocrit (most extreme value from the normal range; if there were no haematocrit measurements available then three times the recorded haemoglobin values were used instead); lowest white blood cell count; lowest total Glasgow Coma Score (GCS); IMV; severe sepsis; cardiopulmonary resuscitation within 24 hours prior to admission; and acute hospital length of stay before ICU admission in days.

Continuous variables were divided into categories for modelling, except for age and hospital length of stay before ICU admission, which were assumed to have a linear effect on the log odds.

#### Evaluation of APACHE II, SAPS II and ICNARC models in haematological malignancy admissions

The SAPS II, the APACHE II score and the ICNARC physiology score were evaluated for discrimination (the ability of the model to distinguish survivors from nonsurvivors), and the APACHE II mortality probability (using coefficients from the model that has been calibrated using the CMPD [25]), the ICNARC model mortality probability and the SAPS II mortality probability were evaluated for discrimination and calibration (the accuracy of the estimated probability of survival). The APACHE II and ICNARC models are used to predict the probability of ultimate acute hospital mortality. The SAPS II model is used to predict the probability of mortality within the same hospital that houses the ICU where the admission occurred.

Discrimination was assessed by the area under the receiver operating characteristic curve (AUROC) [26]. Calibration was assessed by the standardised mortality ratio (SMR), the Hosmer–Lemeshow *C *statistic [27] and Cox's regression calibration [28].

The AUROC (also called the concordance statistic) measures the probability that a randomly selected nonsurvivor has a higher prediction than a randomly selected survivor. A value of 0.5 indicates no discrimination, and a value of 1 indicates perfect discrimination. Values higher than 0.8 are generally considered to demonstrate good discrimination, values between 0.6 and 0.8 are considered moderate, and values lower than 0.6 are considered poor.

The SMR can be used to compare the discrepancy between observed and expected deaths between groups. The ratio is calculated as the number of observed deaths divided by the number of deaths predicted by the model.

The Hosmer–Lemeshow test divides the data into 10 groups and compares the observed mortality in these groups with the expected mortality predicted by the model. The *C *statistic is a chi-squared statistic for testing the hypothesis of perfect calibration (observed mortality = expected mortality). A significant result indicates that calibration is not perfect [27].

Cox's regression calibration tests for a systematic lack of calibration by performing a linear recalibration of the log odds. The log odds are given by log(*p*/(1 - *p*)), where *p *is the mortality probability. The following model is fitted:

If the model is perfectly calibrated then the slope will be 1 and the intercept will be 0; that is, true log odds = predicted log odds. This is tested with a likelihood ratio chi-squared test, with a significant result indicating lack of calibration.

Readmissions within the same acute hospital stay were excluded from all analyses of acute hospital mortality. Patients who stayed less than 8 hours in the ICU were excluded from the calculation of APACHE II scores and probabilities. In addition, patients transferred from another ICU and admissions following coronary artery bypass graft or for primary burns were excluded from the calculation of APACHE II and SAPS II probabilities. In addition, patients were excluded from the calculation of SAPS II probability if no respiratory rates were recorded or no measurements from blood gases were taken. There are no exclusions from the ICNARC model.

All analyses were performed using Stata 9.2 (Stata Corporation, College Station, TX, USA).

## Results

### Case mix

Patients with haematological malignancy accounted for 7,689 admissions (1.5% of all admissions) to ICUs between December 1995 and March 2007. Table 1 presents the characteristics of the patients. Fifty-five per cent of patients were ventilated during the first 24 hours of ICU admission, and 54.3% of patients had a physiological diagnosis of severe sepsis on admission.

**Table 1:** Case mix for admissions with haematological malignancy

	All admissions (n = 7,689)
Age, mean (SD)	57.5 (17.6)
Male, *n *(%)	4,638 (60.3)
APACHE II Acute Physiology Score, mean (SD)	17.1 (7.4)
APACHE II score, mean (SD)	24.4 (7.9)
ICNARC physiology score, mean (SD)	23.7 (11.4)
Number of organ system failures, mean (SD)	1.5 (1.2)
Ventilated at any time during the first 24 hours in the ICU, *n *(%)	4,244 (55.4)
Severe sepsis, *n *(%)	4,177 (54.3)
Physiology	
Lowest platelet count (× 10^9^/l), median (IQR)	74 (31 to 162)
Lowest white blood cell count (× 10^9^/l), median (IQR)	6.7 (2.1 to 14.0)
Outcome	
Mortality, *n *(%)	
Unit	3,312 (43.1)
Hospital^a^	4,239 (59.2)
Activity	
Unit length of stay (days), median (IQR)	
Survivor	2.5 (1.0 to 5.9)
Nonsurvivor	2.2 (0.7 to 6.3)
All	2.3 (0.9 to 6.1)
Hospital length of stay (days)^a^, median (IQR)	
Survivor	27 (14 to 50)
Nonsurvivor	14 (5 to 29)
All	19 (9 to 38)
Transferred in from another ICU, *n *(%)	166 (2.2)
Readmission within the same hospital stay, *n *(%)	449 (5.8)
Hospital mortality by number of organ system failures, mortality (95% CI)	
0 organ failures	33.8 (31.4 to 36.2)
1 organ failure	50.3 (48.3 to 52.4)
2 organ failures	68.3 (66.1 to 70.4)
3 organ failures	83.9 (81.5 to 86.1)
4 organ failures	92.3 (89.3 to 94.6)
5 organ failures	98.8 (93.7 to 99.9)

APACHE, Acute Physiology And Chronic Health Evaluation; APS, acute physiology score; CI, confidence interval; ICNARC, Intensive Care National Audit & Research Centre; ICU: intensive care unit; IQR: interquartile range; SD: standard deviation. ^a^Excluding readmissions within the same hospital stay.

Thrombocytopaenia was present in 4,745 (61.7%) patients, with a median lowest recorded platelet count of 74 × 10^9^/l. Two thousand and twenty-nine (26.4%) patients were leukopaenic on admission.

Case-mix data for admissions by diagnostic category are presented in Table 2. The mean age differs according to the diagnostic category, with a greater mean age in the myeloma, the chronic myelogenous or chronic lymphocytic leukaemia and the non-Hodgkin's lymphoma categories, and lower mean ages in the acute lymphocytic leukaemia and the bone marrow transplant categories. Patients with chronic myelogenous or chronic lymphocytic leukaemia had relatively higher median admission leukocyte counts.

**Table 2:** Case mix for admissions with haematological malignancy by diagnostic category

	AML (n = 622)	ALL (n = 272)	CMLL (n = 310)	
Age, mean (SD)	49.8 (16.8)	33.5 (20.1)	61.7 (16.1)	
Male, *n *(%)	331 (53.2)	143 (52.6)	205 (66.1)	
APACHE II Acute Physiology Score, mean (SD)	20.1 (6.9)	18.6 (6.9)	17.2 (6.9)	
APACHE II score, mean (SD)	26.3 (7.6)	23.8 (7.2)	24.9 (7.6)	
ICNARC physiology score, mean (SD)	26.5 (11.1)	24.1 (11.2)	23.7 (11.4)	
Number of organ system failures, mean (SD)	1.9 (1.2)	1.7 (1.1)	1.5 (1.1)	
Mechanically ventilated at any time during first 24 hours in the ICU, *n *(%)	329 (53.1)	129 (47.6)	165 (53.4)	
Severe sepsis, *n *(%)	284 (45.7)	121 (44.5)	137 (44.2)	
Physiology				
Lowest platelet count (× 10^9^/l), median (IQR)	26 (13 to 51)	34 (19 to 62)	61 (34 to 121)	
Lowest white blood cell count (× 10^9^/l), median (IQR)	2.3 (0.2 to 16.1)	1.9 (0.2 to 6.8)	10.3 (3.1 to 34.6)	
Mortality, *n *(%)				
Unit	341 (54.8)	116 (42.7)	126 (40.7)	
Acute hospital^a^	398 (67.3)	141 (55.5)	165 (56.9)	
Unit length of stay (days), median (IQR)				
Unit survivor	2.8 (1.0 to 5.6)	2.2 (1.0 to 4.9)	2.2 (1.0 to 6.1)	
Unit nonsurvivor	1.5 (0.6 to 4.4)	3.4 (1.0 to 10.2)	1.5 (0.4 to 6.3)	
All	1.9 (0.8 to 5.3)	2.6 (1.0 to 6.7)	1.9 (0.8 to 6.1)	
Acute hospital length of stay (days)^a^, median (IQR)				
Hospital survivor	38 (27 to 62)	39 (21 to 61)	19 (11 to 41)	
Hospital nonsurvivor	15 (3 to 27)	23 (8 to 45)	9 (3 to 24)	
All	22 (8 to 39)	29 (13 to 51)	15 (7 to 33)	
Readmission within the same acute hospital stay, *n *(%)	28 (4.5)	14 (5.2)	18 (5.8)	
Acute hospital mortality by number of organ system failures, *n *(%)				
0 organ failures	24 (47.1)	10 (27.8)	16 (29.6)	
1 organ failure	99 (54.4)	39 (48.2)	51 (45.5)	
2 organ failures	114 (65.9)	49 (60.5)	44 (67.7)	
3 organ failures	105 (85.4)	31 (79.5)	38 (90.5)	
4 organ failures	47 (88.7)	9 (64.3)	15 (93.8)	
5 organ failures	9 (100.0)	3 (100.0)	1 (100.0)	

	Hodgkin's lymphoma (n = 216)	Non-Hodgkin's lymphoma (n = 1,007)	Bone marrow transplant (n = 156)	Myeloma (n = 397)

Age, mean (SD)	50.7 (17.9)	56.3 (15.8)	40.8 (14.7)	63.0 (11.2)
Male, *n *(%)	123 (56.9)	611 (60.7)	83 (53.2)	244 (61.5)
APACHE II Acute Physiology Score, mean (SD)	16.7 (7.0)	17.2 (7.4)	18.5 (8.6)	17.1 (7.5)
APACHE II score, mean (SD)	23.2 (7.5)	24.1 (8.0)	23.4 (9.5)	24.3 (7.9)
ICNARC physiology score, mean (SD)	23.2 (11.0)	24.0 (10.9)	24.0 (12.4)	24.2 (10.8)
Number of organ system failures, mean (SD)	1.5 (1.2)	1.5 (1.2)	1.7 (1.3)	1.7 (1.3)
Mechanically ventilated at any time during first 24 hours in the ICU, *n *(%)	115 (53.5)	528 (52.7)	69 (45.1)	209 (52.8)
Severe sepsis, *n *(%)	101 (46.8)	414 (41.1)	68 (43.6)	146 (36.8)
Physiology				
Lowest platelet count (× 10^9^/l), median (IQR)	75 (29 to 188)	82 (33 to 184)	37 (19 to 62)	95 (47 to 161)
Lowest white blood cell count (× 10^9^/l), median (IQR)	6.1 (2.1 to 10.5)	6.3 (1.8 to 13.6)	3.7 (0.6 to 8.2)	4.7 (1.9 to 8.8)
Mortality, *n *(%)				
Unit	119 (55.1)	492 (48.9)	74 (47.4)	151 (38.0)
Acute hospital^a^	142 (71.0)	625 (66.2)	93 (65.0)	227 (60.1)
Unit length of stay (days), median (IQR)				
Unit survivor	2.7 (1.2 to 5.4)	2.9 (1.3 to 6.7)	2.6 (1.1 to 8.3)	2.9 (1.2 to 8.1)
Unit nonsurvivor	4.5 (1.2 to 8.7)	2.7 (0.9 to 6.4)	3.9 (1.1 to 7.7)	2.8 (0.7 to 7.4)
All	3.4 (1.2 to 7.2)	2.7 (1.1 to 6.4)	3.0 (1.1 to 7.9)	2.9 (1.1 to 7.7)
Acute hospital length of stay (days)^a^, median (IQR)				
Hospital survivor	24 (12 to 44)	29 (15 to 53)	32 (17 to 63)	34 (18 to 55)
Hospital nonsurvivor	15 (6 to 26)	15 (6 to 30)	28 (13 to 46)	14 (5 to 25)
All	17 (8 to 35)	19 (9 to 35)	29 (15 to 54)	20 (9 to 38)
Readmission within the same acute hospital stay, *n *(%)	16 (7.4)	59 (5.9)	11 (7.1)	10 (2.5)
Acute hospital mortality by number of organ system failures, *n *(%)				
0 organ failures	26 (54.2)	90 (43.7)	6 (25.0)	24 (32.4)
1 organ failure	43 (71.7)	182 (60.3)	27 (62.8)	54 (49.1)
2 organ failures	38 (70.4)	197 (75.2)	28 (71.8)	70 (68.0)
3 organ failures	24 (88.9)	102 (87.9)	13 (72.2)	41 (77.4)
4 organ failures	10 (100.0)	41 (93.2)	17 (100.0)	27 (100.0)
5 organ failures	1 (100.0)	13 (92.9)	2 (100.0)	11 (100.0)

Only admissions with a haematological diagnosis as the primary, secondary or ultimate reason for admission are included. The numbers with each diagnosis are greater than in the logistic regression model because admissions missing hospital outcome and readmissions are excluded from the regression analysis. AML: acute myelogenous leukaemia; ALL: acute lymphocytic leukaemia; APACHE: Acute Physiology and Chronic Health Evaluation; APS: Acute Physiology Score; CI: confidence interval; CMLL: chronic myelogenous or chronic lymphocytic leukaemia; ICNARC: Intensive Care National Audit & Research Centre; ICU: intensive care unit; IQR: interquartile range; SD: standard deviation. ^a^Excluding readmissions within the same acute hospital stay.

### Outcome and activity

Overall 3,312 (43.1%) patients died in intensive care and 4,239 (59.2%) died during the hospital admission (Table 1). The median length of stay on the ICU was 2.3 days, survivors having a slightly longer median stay than nonsurvivors. Four hundred and forty-nine (5.8%) patients were readmitted to the ICU during the same hospital admission, and 166 (2.2%) were transferred from another ICU.

### Effect of organ failure on survival

As the number of organ failures present on admission increased, there was an increase in hospital mortality (Table 1). If five organ failures were present, the hospital mortality was 98.8%.

### Prognostic ability of the SAPS II, APACHE II and ICNARC models

**Figure 1:**
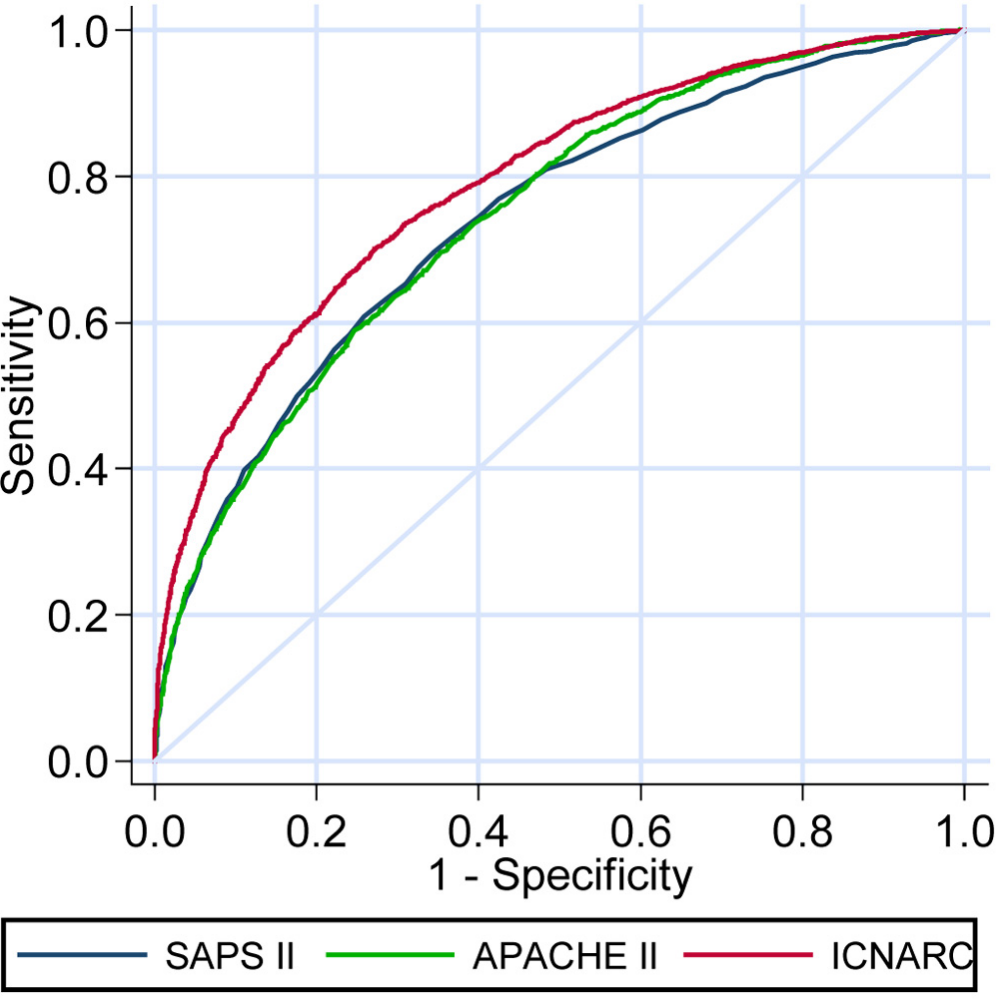
Receiver operating characteristic curves for the SAPS II, APACHE II and ICNARC physiology scores.

The discrimination and calibration of the SAPS II, APACHE II and ICNARC scores are presented in Table 3. The three models all showed reasonably good discrimination between survivors and nonsurvivors as assessed by the AUROC (Figure 1), with the ICNARC model (AUROC = 0.79) demonstrating slightly better discrimination than the APACHE II and SAPS II models (AUROC = 0.74).

**Table 3:** Model fit – comparison of the SAPS II, APACHE II and ICNARC models

	SAPS II model	APACHE II model	ICNARC model
Eligible admissions, *n *(%)	4,973 (64.6)	6,212 (80.7)	7,156 (93.1)
Observed deaths	3,030	3,579	4,237
Expected deaths	2,675.2	3,538.5	3,393.3
SMR (95% CI)	1.13 (1.11 to 1.16)	1.01 (0.99–1.03)	1.25 (1.22 to 1.27)
AUROC (95% CI)	0.74 (0.73 to 0.75)	0.74 (0.73 to 0.76)	0.79 (0.78 to 0.80)
Hosmer–Lemeshow *C*^a ^statistic			
χ^2^(10)	568.0	68.0	900.9
*P *value	< 0.001	< 0.001	< 0.001
Cox's calibration regression			
Intercept (95% CI)	0.44 (0.38 to 0.51)	0.08 (0.02 to 0.14)	0.62 (0.56 to 0.67)
Slope (95% CI)	0.59 (0.54 to 0.63)	0.80 (0.75 to 0.85)	0.77 (0.73 to 0.81)
χ^2^(2)	469.4	54.3	708.95
*P *value	< 0.001	< 0.001	< 0.001

The Hosmer-Lemeshow test divides the data into ten groups and compares the observed mortality in these groups to the predicted mortality given by the model. The *C *statistic is a chi-squared statistic for testing the hypothesis of perfect calibration. A significant value for the *C *statistic indicates that calibration is not perfect.

APACHE II: Acute Physiology and Chronic Health Evaluation II; AUROC: area under the receiver operating characteristic curve; CI: confidence interval; ICNARC: Intensive Care National Audit and Research Centre; SAPS II: Simplified Acute Physiology Score II; SMR: standardised mortality ratio. ^a^

**Figure 2:**
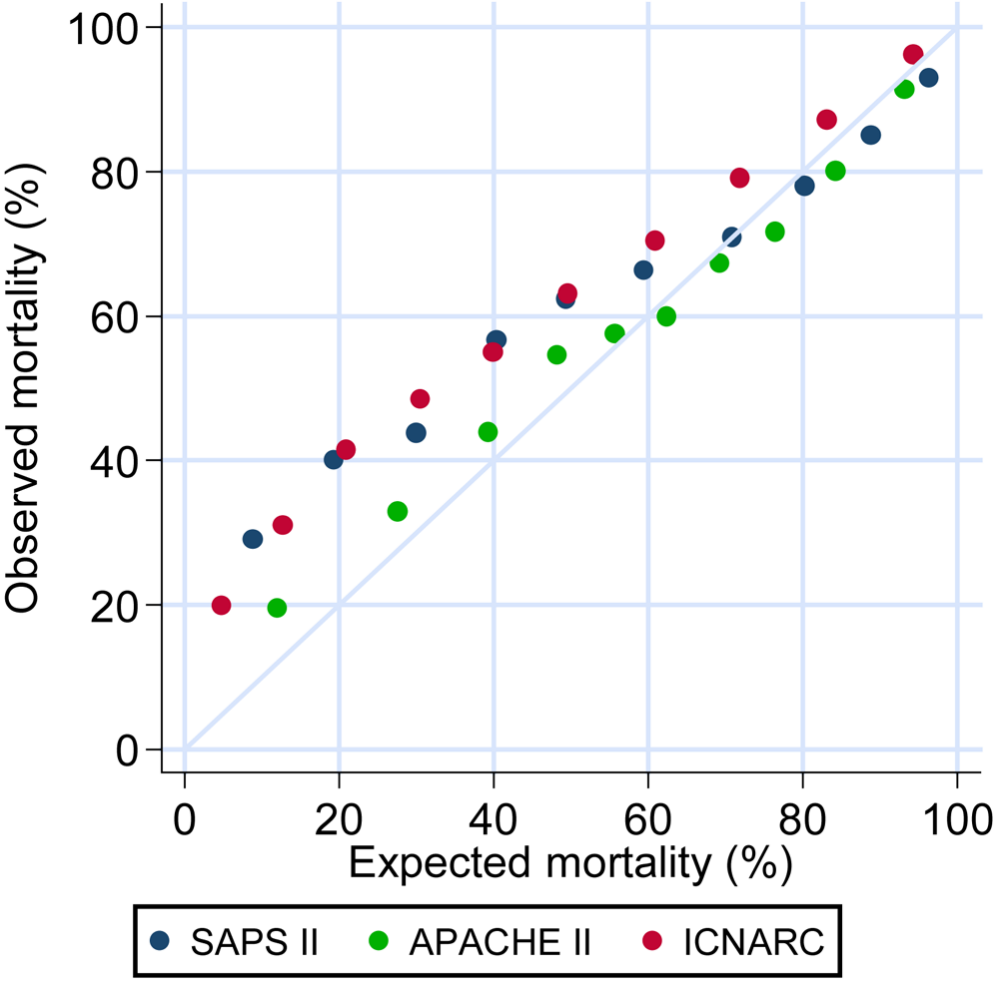
Calibration plot of SAPS II, APACHE II and ICNARC physiology scores. A comparison of the goodness-of-fit of the SAPS II, APACHE II and ICNARC physiology scores.

The APACHE II model gave the best prediction of actual mortality, with an SMR of 1.01. The SAPS II (SMR = 1.13) and ICNARC models (SMR = 1.25), however, considerably underestimated hospital mortality. The calibration of all three models, as assessed by the Hosmer–Lemeshow goodness-of-fit *C *statistic and Cox's calibration regression, was poor. The APACHE II model was better calibrated than either the SAPS II model or the ICNARC model. All three models underestimated actual mortality when the predicted mortality was low (Figure 2), although the APACHE II model lies closer to the line of perfect fit than the SAPS II model or the ICNARC model, indicating that it had better calibration than the other two models when the predicted mortality was low.

### Factors associated with acute hospital mortality

The results of multiple logistic regression analysis are summarised in Tables 4 and 5.

**Table 4:** Multivariate logistic regression analysis for admissions to critical care units with haematological malignancy^a^

	Number of admissions	Number of deaths	Percentage of deaths	Adjusted	
				
				Odds ratio (95% CI)	*P *value
Age					< 0.001
<45 years	1,512	831	55.0	1.14 (1.09 to 1.20)	
45 to 54 years	1,053	622	59.1	Per 10-year increase	
55 to 64 years	1,716	1,006	58.6		
65 to 74 years	1,852	1,171	63.2		
75+ years	1,028	609	59.2		
Sex					0.742
Female	2,818	1,709	60.7	1.02 (0.89 to 1.18)	
Male	4,343	2,530	58.3	Reference	
Diagnostic category^b^					< 0.001
Bone marrow transplant	143	93	65.0	1.88 (1.00 to 3.53)	
ALL	254	141	55.5	Reference	
AML	591	398	67.3	1.37 (0.86 to 2.20)	
CLML	290	165	56.9	1.02 (0.58 to 1.80)	
Hodgkin's lymphoma	200	142	71.0	2.38 (1.30 to 4.36)	
Non-Hodgkin's lymphoma	944	625	66.2	1.46 (0.92 to 2.31)	
Myeloma	378	227	60.1	0.79 (0.47 to 1.35)	
Highest central temperature					0.100
<36.0°C	127	110	86.6	2.26 (0.97 to 5.29)	
36.0 to 38.4°C	3,850	2,205	57.3	Reference	
38.5 to 40.9°C	2,900	1,714	59.1	0.94 (0.81 to 1.09)	
41.0+°C	121	80	66.1	0.70 (0.41 to 1.20)	
Lowest systolic blood pressure					< 0.001
<50 mmHg	281	265	94.3	3.66 (1.93 to 6.96)	
50 to 59 mmHg	303	251	82.8	1.66 (1.09 to 2.54)	
60 to 69 mmHg	601	472	78.5	1.55 (1.15 to 2.08)	
70 to 79 mmHg	1,005	667	66.4	1.10 (0.88 to 1.37)	
80 to 99 mmHg	2,598	1,493	57.5	1.14 (0.97 to 1.34)	
100+ mmHg	2,270	1,018	44.9	Reference	
Highest heart rate					< 0.001
<110 beats/min	1,895	788	41.6	Reference	
110 to 119 beats/min	1,031	553	53.6	1.32 (1.06 to 1.64)	
120 to 139 beats/min	2,119	1,326	62.6	1.57 (1.30 to 1.90)	
140+ beats/min	2,019	1,503	74.4	2.07 (1.67 to 2.55)	
Highest respiratory rate, ventilated or nonventilated					< 0.001
<6 breaths/min	217	141	65.0	1.03 (0.69 to 1.53)	
6 to 11 breaths/min	1,840	893	48.5	Reference	
12 to 13 breaths/min	1,623	1,039	64.0	1.36 (1.12 to 1.65)	
14 to 24 breaths/min	2,899	1,743	60.1	1.57 (1.31 to 1.87)	
25+ breaths/min	468	344	73.5	2.81 (2.00 to 3.95)	
PaO_2_:FiO_2_					< 0.001
Ventilated					
<100 mmHg	580	386	66.6	2.78 (2.11 to 3.66)	
100 to 199 mmHg	888	446	50.2	1.34 (1.07 to 1.69)	
200+ mmHg	1,162	441	38.0	Reference	
Nonventilated					
<100 mmHg	1,220	1,033	84.7	2.71 (1.45 to 5.06)	
100 to 199 mmHg	1,374	951	69.2	1.33 (0.72 to 2.45)	
200+ mmHg	1,108	621	56.1	1.06 (0.58 to 1.95)	
Lowest arterial pH					< 0.001
<7.15	1,248	1,125	90.1	2.25 (1.71 to 2.96)	
7.15 to 7.24	1,129	817	72.4	1.37 (1.11 to 1.70)	
7.25 to 7.32	1,457	797	54.7	1.09 (0.91 to 1.30)	
7.33 to 7.49	2,453	1,094	44.6	Reference	
7.50+	93	61	65.6	2.20 (1.24 to 3.92)	
Serum sodium					<0.001
<130 mmol/l	285	208	73.0	2.47 (1.70 to 3.60)	
130 to 149 mmol/l	6,125	3,486	56.9	Reference	
150 to 154 mmol/l	248	196	79.0	1.85 (1.25 to 2.73)	
155 to 159 mmol/l	72	63	87.5	3.28 (1.45 to 7.40)	
160+ mmol/l	36	28	77.8	1.34 (0.52 to 3.46)	

^a^Analysis of the effect of age, gender, diagnostic category, temperature, systolic blood pressure, heart rate, respiratory rate, PaO_2_:FiO_2_, arterial pH and serum sodium on outcome. ALL: acute lymphoblastic leukaemia; AML: acute myeloblastic leukaemia; CI: confidence interval; CLML: chronic lymphocytic or myeloblastic leukaemia. ^b^Only admissions with a haematological diagnosis as the primary, secondary or ultimate reason for admission were included in this analysis.

**Table 5:** Multivariate logistic regression analysis for admissions to critical care units with haematological malignancy^a^

	Number of admissions	Number of deaths	Percentage of deaths	Adjusted	
				
				Odds ratio (95% CI)	*P *value
Serum potassium					
<3.0 mmol/l	63	45	71.4	2.40 (1.02 to 5.67)	0.226
3.0 to 3.4 mmol/l	274	169	61.7	1.29 (0.87 to 1.92)	
3.5 to 5.4 mmol/l	5,548	3,124	56.3	Reference	
5.5 to 5.9 mmol/l	431	303	70.3	0.93 (0.69 to 1.27)	
6.0 to 6.9 mmol/l	343	260	75.8	1.12 (0.79 to 1.61)	
7.0+ mmol/l	106	83	78.3	0.79 (0.40 to 1.53)	
Serum urea					<0.001
<6.2 mmol/l	1,315	504	38.3	1.05 (0.78 to 1.42)	
6.2 to 7.1 mmol/l	422	189	44.8	Reference	
7.2 to 14.3 mmol/l	2,158	1,272	58.9	1.45 (1.09 to 1.93)	
14.4+ mmol/l	2,283	1,666	73.0	2.39 (1.74 to 3.28)	
Serum creatinine					0.310
<0.6 μmol/l	193	94	48.7	1.41 (0.94 to 2.13)	
0.6 to 1.4 μmol/l	3,104	1,510	48.7	Reference	
1.5+ μmol/l	3,359	2,302	68.5	0.84 (0.70 to 1.02)	
Urine output					< 0.001
<400 ml/day	1,121	956	85.3	2.91 (2.23 to 3.79)	
400 to 599 ml/day	297	239	80.5	2.66 (1.76 to 4.00)	
600 to 899 ml/day	488	341	69.9	2.05 (1.53 to 2.75)	
900 to 1,499 ml/day	1,270	752	59.2	1.58 (1.30 to 1.91)	
1,500 to 1,999 ml/day	984	515	52.3	1.37 (1.13 to 1.66)	
2,000+ ml/day	2,689	1,229	45.7	Reference	
Haematocrit					0.001
<20%	171	119	69.9	3.22 (0.96 to 10.85)	
20 to 29%	3,073	1,904	62.0	4.56 (1.51 to 13.80)	
30 to 45%	3,405	1,897	55.7	3.69 (1.22 to 11.13)	
46 to 49%	55	28	50.9	2.13 (0.58 to 7.82)	
50 to 59%	26	10	38.5	Reference	
60+%	5	3	60.0	1.24 (0.09 to 17.20)	
Lowest white blood cell count					0.425
<1 × 10^9^/l	1,217	799	65.7	1.17 (00.95 to 1.45)	
1 to 2 × 10^9^/l	820	503	61.3	1.15 (0.92 to 1.43)	
3 to 14 × 10^9^/l	3,009	1,605	53.3	Reference	
15 to 39 × 10^9^/l	1,039	616	59.3	0.98 (0.80 to 1.20)	
40+ × 10^9^/l	459	301	65.6	1.16 (0.86 to 1.56)	
Glasgow Coma Score					< 0.001
3	511	437	85.5	3.32 (2.33 to 4.74)	
4	29	21	72.4	1.58 (0.55 to 4.52)	
5	30	22	73.3	2.20 (0.85 to 5.73)	
6	81	54	66.7	1.47 (0.80 to 2.71)	
7 to 13	595	392	65.9	1.80 (1.40 to 2.31)	
14	376	223	59.3	1.48 (1.11 to 1.98)	
15	3,190	1,385	43.4	Reference	
Sedated	1,518	1,119	73.7	1.85 (1.50 to 2.28)	
Sedated and paralysed	214	173	80.8	2.40 (1.51 to 3.82)	
Mechanical ventilation					0.154
No	3,169	1,435	45.3	Reference	
Yes	3,970	2,788	70.2	1.53 (0.85 to 2.73)	
Severe sepsis					0.001
No	3,245	1,630	50.2	Reference	
Yes	3,916	2,609	66.6	1.29 (1.10 to 1.50)	
CPR within 24 hours prior to admission					0.317
No	6,728	3,884	57.7	Reference	
Yes	426	353	82.9	1.21 (0.84 to 1.74)	
Hospital length of stay before ICU admission					< 0.001
0 days	1,358	735	54.1	1.02 (1.01 to 1.02)	
1 day	1,266	614	48.5	(per day)	
2 to 4 days	1,253	690	55.1		
5 to 9 days	977	617	63.2		
10 to 19 days	1,109	735	66.3		
>20 days	1,195	846	70.8		

^a^Analysis of the effect of serum potassium, urea, creatinine, urine output, haematocrit, white cell count, Glasgow Coma Score, invasive ventilation, severe sepsis, cardiopulmonary resuscitation (CPR) and length of hospital stay prior to intensive care admission on outcome. CI: confidence interval; ICU: intensive care unit.

Nineteen factors were found to be associated with acute hospital mortality. Patients with severe sepsis had a higher risk of acute hospital mortality (adjusted odds ratio (OR) = 1.29). There was also an increase in mortality with increasing age, with an adjusted OR of 1.14 for every 10-year increase in age. As the time interval between acute hospital admission and admission to intensive care increased, the acute hospital mortality also increased. Acute hospital mortality was 54.1% in patients immediately admitted to the ICU, compared with 70.8% if admission occurred after 20 days or more in hospital.

Other factors found to be associated with an increased risk of hospital mortality were haematocrit, systolic blood pressure, respiratory rate, heart rate, GCS, sedation, PaO_2_:, arterial pH, urine output, serum sodium, and serum urea. Of these factors, haematocrit from 20 to 29% (adjusted OR = 4.56), systolic hypotension <50 mmHg (adjusted OR = 3.66), and a GCS of 3 (adjusted OR = 3.32) conferred the highest odds for hospital mortality.

Although IMV within 24 hours of ICU admission was not associated with hospital mortality after adjustment for other prognostic factors, 70.2% of intubated patients died compared with 45.3% of nonintubated patients.

Two thousand eight hundred admissions were included in the subgroup analysis of the effect of diagnosis on outcome. Admission after HSCT (mortality 65%, adjusted OR = 1.88) or admission with a diagnosis of Hodgkin's lymphoma (mortality 71%, adjusted OR = 2.38) were both associated with higher hospital mortality.

## Discussion

The acute hospital mortality of patients with haematological malignancies admitted to adult, general ICUs in England, Wales and Northern Ireland between 1995 and 2007 was 59.2%. Factors present on admission associated with increasing acute hospital mortality included age, length of hospital stay prior to admission and the presence of severe sepsis. Patients with Hodgkin's lymphoma and those who had received HSCT had an increased risk of death.

We compared the performance of the SAPS II, APACHE II and ICNARC models in predicting mortality. The ICNARC model had the best discrimination as assessed by the AUROC, but significantly underestimates mortality; while the APACHE II model does not underestimate mortality for this group of patients, but has slightly less discriminative power than the ICNARC model. None of the models showed good calibration as assessed by the Hosmer–Lemeshow goodness-of-fit test or Cox's calibration regression.

### Strengths of the study

The present trial is a large study assessing the outcome of admissions with haematological malignancies to the ICU, including data from 178 different units. The generalisability of our results to similar patient populations is enhanced by the number of units contributing data. The CMPD is recognised to be of high quality, meaning that the data used in this study are reliable.

### Limitations of the study

The CMPD was not primarily designed to analyse the outcome of critically ill patients with haematological malignancies, imposing important limitations on the study. Only routinely collected admission data included in the CMPD were available for analysis. Therefore, it was not possible to analyse all of the prognostic factors in haematological malignancy patients, such as neutropaenia, or the type of HSCT received. We could not determine whether patients had received allogeneic or autologous HSCT, or peripheral blood stem cell transplants prior to ICU admission, where the mortality of patients receiving these treatments is known to differ. Although we analysed the effect of leukopaenia on outcome, this is not the same as neutropaenia. The analysis of the effect of haematological diagnosis on outcome was also limited by the grouping of different haematological conditions in the past medical history field of the CMPD. We therefore performed a subgroup analysis on 2,800 admissions where a precise haematological diagnosis could be extracted. We are also unable to analyse the effect of physiology on outcome beyond the first 24 hours of ICU stay, where deteriorating physiology may be useful in making decisions regarding withholding or withdrawing treatment. We are not able to report on long-term follow up for mortality in these admissions.

It is likely that this retrospective study was subject to inclusion bias. Differences in admission policies among the contributing units may have led to the inclusion of data from patients with incurable illness who were certain to die, and to the exclusion of critically ill patients with haematological malignancy who were managed on the ward, of whom there are a large number [6]. Eligible patients may have been omitted, or ineligible patients included. For example, HSCT may be used to treat some solid tumours and autoimmune disorders, as well as haematological malignancies. The proportion of HSCT patients, however, was small (143 out of 7,689 admissions) – the majority of whom would have had a haematological malignancy.

During the time period covered by the present study, several novel therapies for critically ill haematological malignancy patients have been introduced, such as noninvasive ventilation and recombinant colony-stimulating factors. The use of these therapies may have improved the survival of many patients included in this study [29]. In addition, the management of sepsis has been improved by the introduction of the Surviving Sepsis Campaign in 2002. It has been suggested that cancer patients with severe sepsis have a better chance of survival than in the past [30,31]. Despite this, the unit and hospital mortality rates of patients in this study did not change significantly over time (data not shown). Finally, lead-time bias may have affected the results. Lead-time bias results from patients' physiology improving as they receive therapy usually provided in the ICU while awaiting ICU admission.

#### Predictive power of the APACHE II, SAPS II and ICNARC models

The SAPS II and APACHE II models have been assessed in small studies of critically ill haematological malignancy patients. The AUROC for SAPS II was found to be 0.765 [32] and 0.78 [12]. Benoit and colleagues found that the SAPS II model had better discrimination than the APACHE II model (AUROC = 0.712) [32]; however, both studies included only small numbers of patients. Both the ICNARC and SAPS II models underestimated actual mortality in this study. Underestimation of mortality in haematological malignancy patients by the SAPS II model has been previously described [6,11]. The APACHE II model has also been found to underestimate mortality in these patients [8,11], but we found that the APACHE II model gave the most accurate mortality prediction (Table 3) with an SMR of 1.01.

### Effect of organ failure on survival

As the number of organ failures present on admission increased, the hospital mortality also increased; less than 10% of patients with four organ failures survived, and hospital mortality was 98.8% when there were five organ failures present on admission (Tables 1 and 2).

### Risk factors for hospital mortality

Previous studies have identified risk factors for hospital mortality following admission to the ICU with a haematological malignancy (Table 6). We were able to assess the effects of haematological diagnosis, HSCT, hospital length of stay, age, admission physiology, IMV, and severe sepsis within the first 24 hours of admission.

**Table 6:** Risk factors for mortality in patients admitted to intensive care with haematological malignancy

Study	Factors identified that are associated with hospital mortality
Owzcuk and colleagues [16]	High SAPS II, SOFA score or APACHE II score, neutropaenia, hypotension, cardiovascular failure
Yau and colleagues [9]	Progression of underlying malignancy
Massion and colleagues [11]	Respiratory failure, fungal infection, number of organ failures, haemopoeitic stem cell transplant status
Benoit and colleagues [32]	Leukopaenia, use of vasopressors, urea >0.75 g/l
Gordon and colleagues [6]	Hepatic failure, central nervous system failure, SAPS II score >66
Lim and colleagues [39]	Raised bilirubin, inotropic support, more than three organ failures
Lloyd-Thomas and colleagues [8]	High APACHE II score, number of organ failures, mechanical ventilation
Brunet and colleagues [10]	SAPS II score, more than one organ failure
Kroschinsky and colleagues [14]	Haemofiltration, high SAPS II score
Silfvast and colleagues [50]	Maximal SOFA score on admission
Cornet and colleagues [17]	SAPS II and SOFA score over 96 hours
Lamia and colleagues [12]	Severe sepsis or septic shock, vasopressor use, invasive ventilation, neutropaenia, allogeneic haemopoeitic stem cell transplant, high SAPS II, SOFA score, ODIN score or LODS score
Bianchi and colleagues [41]	Mechanical ventilation, neutropaenia, renal failure

APACHE II: Acute Physiology and Chronic Health Evaluation II; LODS: Logistic Organ Dysfunction Score; ODIN: Organ Dysfunction and/or Infection score; SAPS II: Simplified Acute Physiology Score II; SOFA: Sequential Organ Failure Assessment.

#### Haematological diagnosis

We found that patients diagnosed with Hodgkin's lymphoma had an increased risk of hospital mortality (adjusted OR = 2.38). This is an unexpected finding, since patients with Hodgkin's lymphoma have a relatively good long-term prognosis. Groeger and colleagues found in cancer patients receiving IMV that those with leukaemia had a higher mortality [33]. Massion and colleagues found that acute myeloid leukaemia and non-Hodgkin's lymphoma had a negative effect on 6-month survival [11]. In the present study, patients with acute myeloid leukaemia had a higher mortality than those with acute lymphocytic leukaemia, perhaps partially explained by the difference in mean age between these admissions (Table 2). Patients with chronic lymphocytic leukaemia had the best prognosis in the study by Peters and colleagues [7]. Other researchers, however, have found that the type of haematological malignancy was not predictive for mortality [13,16].

#### Bone marrow transplant

Admission after bone marrow transplantation (HSCT) was a risk factor for acute hospital mortality in the present study. The hospital mortality in patients who become critically ill after HSCT is 54 to 67% [1,34,35], with higher mortality rates reported in intubated patients [3,16,36,37]. Patients who receive autologous HSCT transplants undergo less intensive preparative regimens and faster marrow engraftment, so a lower mortality would be expected. Khassawneh and colleagues found that the hospital mortality rate in such patients who were intubated was 74% [38]. Indeed, allogeneic HSCT has been identified as a risk factor for mortality [33,34]. Other studies, however, have found that the type of HSCT has no influence on mortality [3,35,39]. Dividing HSCT patients into those receiving autologous and allogeneic transplants is not possible in the present study, as the type of HSCT received is not recorded in the CMPD.

#### Admission physiology

Tachypnoea, a low PaO_2_:FiO_2 _and hypotension were associated with increased hospital mortality. The relationship between respiratory failure and increased risk of mortality has already been demonstrated [11]. Requirement for vasopressors has also been associated with a worse outcome in these patients [12,32,39].

In the present study oliguria, hyponatraemia and uraemia correlated with mortality. These abnormalities may result from acute kidney injury, which is strongly associated with mortality in haematological malignancy patients [14,32].

#### Arterial pH

Acidaemia was associated with lower survival. Acute kidney injury or high serum lactate may cause acidosis, and both conditions predicted higher mortality in previous studies [1,14,32,40,41].

#### Invasive mechanical ventilation

Most studies report a strong relationship between IMV and increased mortality [8,12,41]. Using noninvasive ventilation rather than IMV reduces mortality in immunocompromised patients [42,43]. IMV during the first 24 hours of admission was not associated with an increased risk of mortality in this study, although a large absolute difference in mortality exists between patients who received IMV and those who did not.

Depudyt and colleagues found that IMV within 24 hours of ICU admission was related to a *lower *mortality rate in haematological malignancy patients with respiratory failure [44]. Considering those patients who had PaO_2_:FiO_2 _lower than 200 mmHg in the present study, a similar conclusion may be drawn, as those who were invasively ventilated within 24 hours had lower mortality rates than those who were not. Indeed, we found that severe respiratory failure (PaO_2_:FiO_2 _< 100 mmHg) without IMV is associated with increased hospital mortality. This may reflect reluctance to use IMV in such patients, as less than one-third of patients who had a PaO_2_:FiO_2 _< 100 mmHg received IMV (Table 5).

#### Leukopaenia

We did not find an association between leukopaenia and mortality, although in previous studies leukopaenia was identified as a significant risk factor [8,32] and leukopaenia is a component of the APACHE II score and the SAPS II. We were unable to assess the effect of neutropaenia on mortality, as this is not a recorded parameter in the CMPD.

#### Glasgow Coma Score

A low GCS was independently associated with mortality. Gordon and colleagues [6] found that central nervous system failure was associated with higher mortality.

The retrospective nature of the present study should lead to caution in interpreting the factors identified as being associated with mortality. Severe sepsis – together with cardiorespiratory and renal dysfunction – was associated with increased mortality, however, and this implies that early, aggressive treatment aimed at reversing organ dysfunction may improve survival, particularly in those patients with severe sepsis.

We also found that acute hospital mortality was associated with a longer stay in hospital prior to ICU admission. This is probably due to delayed recognition and suboptimal treatment of patients who have become acutely unwell on the ward, suggesting that earlier admission to the ICU may benefit some patients. Groeger and colleagues found a correlation between hospital length of stay prior to ICU admission and mortality in critically ill cancer patients [33,45,46], and this has also been found in critically ill patients as a whole [47].

A low haematocrit of between 20% and 45% was associated with higher mortality, compared with admissions with a haematocrit of between 50% and 59. Anaemia has not previously been associated with an increased risk of hospital mortality in critically ill patients with haematological malignancy. Current practice in intensive care patients is to tolerate a fall in haematocrit, excepting certain groups of patients, based on evidence that this practice is not harmful [48]. On the other hand, in the early phase of severe sepsis, targeting a haematocrit of at least 30% is associated with improved survival [49]. In patients with a haematological malignancy a low haematocrit is common, and its causes are multifactorial. The low haematocrit may be chronic (resulting from bone marrow suppression or more aggressive chemotherapy) or acute (secondary to haemorrhage). One interpretation of our results would suggest that if haematology malignancy patients were managed with a higher haematocrit, at least initially, then their mortality may be reduced.

The association between hospital mortality and sedation may be partially explained by confounding with the adverse effect on mortality of mechanical ventilation. The relationship between hospital mortality and anaemia on admission in these patients is surprising given previous evidence of safety in tolerating a low haematocrit in intensive care patients. Further studies should be conducted to refute or confirm this relationship.

## Conclusions

The ICU mortality for haematological malignancy patients in this study was 43.1% and the hospital mortality was 59.2%. Admission factors associated with an increased risk of death were severe sepsis, age, bone marrow transplant, Hodgkin's lymphoma, length of hospital stay prior to intensive care admission, tachycardia, systolic blood pressure <70 mmHg, tachypnoea, GCS, sedation, PaO_2_:FiO_2_, acidaemia, alkalaemia, oliguria, hyponatraemia, hypernatraemia, low haematocrit, and uraemia. In contrast to previous studies, we did not find that leukopaenia was associated with increased hospital mortality. We did, however, find that a low haematocrit was associated with higher hospital mortality. This finding warrants further investigation in a prospective study.

The ICNARC model had the best discrimination of the three scores analysed, with an AUROC of 0.78, but all scores were poorly calibrated. The APACHE II model had the highest accuracy at predicting hospital mortality, with a SMR of 1.01. The SAPS II and the ICNARC score both underestimated hospital mortality.

Increased hospital mortality is associated with both increasing length of hospital stay prior to ICU admission and severe sepsis, suggesting that, if appropriate, such patients should be admitted to the ICU early and treated aggressively.

## Key messages

• The ICU mortality for patients with haematological malignancy in this study was 43.1%, and the hospital mortality was 59.2%.

• All of the severity-of-illness scores tested showed poor calibration for mortality, underestimating actual mortality when the predicted mortality was low.

• Increased hospital mortality is associated with increasing length of hospital stay before ICU admission in patients with haematological malignancy.

• Severe sepsis on ICU admission is associated with a poorer outcome in patients with haematological malignancy.

• A low haematocrit on admission to the ICU is also associated with a poor outcome in patients with haematological malignancies.

## Abbreviations

APACHE II: Acute Physiology and Chronic Health Evaluation II; AUROC: area under the receiver operating characteristic curve; CMPD: Case Mix Programme Database; GCS: Glasgow Coma Score; HSCT: haemopoeitic stem cell transplant; ICNARC: Intensive Care National Audit and Research Centre; ICU: intensive care unit; IMV: invasive mechanical ventilation; OR: odds ratio; SAPS II: Simplified Acute Physiology Score II; SMR: standardised mortality ratio.

## Competing interests

The authors declare that they have no competing interests.

## Authors' contributions

PAH wrote the Introduction, Results and Discussion. CAW wrote the Materials and methods. KF contributed to the Results. CAW and DAH performed the statistical analyses and advised on the results of multiple regression analysis. DAH and LAM reviewed the final manuscript.
